# Crucial Roles of LncRNAs-Mediated Autophagy in Breast Cancer

**DOI:** 10.7150/ijms.72621

**Published:** 2022-06-21

**Authors:** Xinwei Han, Jinggang Mo, Yingmei Yang, Yichao Wang, Hongsheng Lu

**Affiliations:** 1 Tai Zhou Central Hospital (Taizhou University Hospital), No.999 Donghai Road, Jiaojiang District, Taizhou, Zhejiang, 318000, China.; 2 Cytotherapy Laboratory, Shenzhen People's Hospital, 1017, Dongmen North Road, Luohu, Shenzhen, 518020, China.

**Keywords:** LncRNA, Autophagy, Molecular mechanisms, Breast cancer, Drug resistance

## Abstract

Breast cancer remains a worldwide public health issue. LncRNA and autophagy respectively or simultaneously, get involved in cellular and molecular processes of many different cancers, including genesis, metastasis, and deterioration of breast cancer and other malignant tumors. In this review, relevant studies have been summarized, and we have found that lncRNA-mediated autophagy in luminal A breast cancer, luminal B breast cancer, HER-2 positive breast cancer, and basal-like breast cancer may play an important role in mediating drug resistance sensitivity. LncRNAs target genes and affect different signaling pathways to a complex network, which attenuates the occurrence and development of primary breast cancer by coordinating autophagy. Abnormal expression of LncRNA may lead to dysregulation of autophagy, resulting in tumor genesis, expansion, and resistance to anti-tumor therapy. Targeting specific lncRNAs for autophagy regulation may conduct as a bio-marker for reliable diagnosis and prognosis treatment of breast cancer or provide a promising therapeutic strategy.

## Introduction

Breast cancers basically include luminal A breast cancer, luminal B breast cancer, HER-2 positive breast cancer, and basal-like breast cancer. According to GLOBCAN, 2020, the morbidity and mortality of breast cancer are separately reported at 11.7% and 6.9% 1. Breast cancer is one of the most common malignancies and the first inducement of cancer-associated death among female subjects in the world. It is reported that in 2020 breast cancer has already been the 5^th^ most cancer worldwide 1.

As one of the most frequently diagnosed cancers among females, breast cancer represents 7%-10% of all malignant tumors. It is predicted that the incidence of estrogen receptor-positive cancers may elevate substantially amid a growing. The statistics of 2020 showed that in the United States the annual spending on the treatment of breast cancer was projected to reach $22.6 billion 2. Although with the development of screening technology, early breast cancer can be screened through mammography technology, for early prevention and treatment, such as surgery and chemoradiotherapy 3, especially in patients who are in advanced and metastatic conditions, are still served as the leading causes of death. Moreover, because of the high resistance to chemotherapy or radiotherapy, unresectable patients with advanced-stage of tumors often have a poor prognosis.

Breast cancer is classified to determine treatment options based on the following molecular markers: The expression of estrogen receptor (ER), progesterone receptor (PR), human epidermal growth factor-2 (HER-2), and Ki-67 can be divided into the following four subtypes 4: Luminal A (ER^+^ and/or PR^+^ and HER2^-^, low expression of Ki67) and Luminal B (ER^+^ and/or PR^+^ and HER2^-^/HER2^+^, high expression of Ki67) 5 accounted for 40% and 30% of cases, respectively, and were the most common types of breast cancer. About 15% of breast cancers are HER2-enriched subtypes with ER^-^/PR^-^/HER2^+^ phenotypes 6. HER2-enriched breast cancer shows increased expression of proliferative markers as well and has a worse prognosis than luminal subtypes 7.

Basal-like breast cancer, also named triple-negative breast cancer (TNBC) (ER^-^/PR^-^/HER2^-^), has the worst prognosis of all the breast cancer types 8, 9.

The molecular mechanism of breast cancer can be a prerequisite for the diagnosis, therapeutic treatment, and prognosis of this cancer. It is urgent to increase the sensitivity of chemotherapy for breast cancer. Apoptosis metabolism disorder plays a key role in the occurrence and development of various malignancies including breast tumors. More and more studies have shown that lncRNA can regulate autophagy to promote apoptosis and thus attenuate the growth of primary tumors 10. In this review, we hope to describe the role of the lncRNA-autophagy axis in the progression and drug resistance of human breast cancer through a systematic review of relevant literature.

## Overview of Autophagy

Autophagy is the major intracellular digestion system and dynamic system which provides energy and new building components for cellular renewal and homeostasis. So far there are roughly three classes of autophagy that includes macroautophagy, microautophagy, and chaperone-mediated autophagy 11. Compared with microautophagy and chaperone-mediated autophagy, the study of macroautophagy has been most extensive and particularly macroautophagy, which is thought to be the main type of autophagy 12, 13. Autophagy, usually regulated by autophagy-related genes (ATGs), is the process by which cell contents are transported to lysosomes for degradation by a two-membrane vesicle structure called an autophagosome 12. Autophagy often plays different roles due to different trigger signals 14. In some cases, autophagy protects cells from death by removing damaged organelles in a self-constructed stress environment and induces cell death when stimulated by other signals 15. Similarly, autophagy plays a complex role in cancer cells, promoting or inhibiting cancer through different molecular mechanisms. Generally, it is believed that autophagy plays a protective role in the progression of many different kinds of tumors by preventing the toxic accumulation of damaged cellular materials in the early stage of tumor development; in advanced stages, autophagy boosts metabolism to meet cancer's high nutritional requirements and keep tumor cells alive 16.

## The role of autophagy in Breast Cancer

There is growing evidence that dysregulation of autophagy can cause a variety of diseases. In 1999, Levine *et al.*[17]explained that the autophagy gene becline1 regulates the development of breast cancer, first revealing the relationship between autophagy and breast cancer. However, from current studies, the exact role of autophagy in breast cancer is still controversial. It has been suggested that autophagy promotes breast cancer progression by increasing the survival rate of latent breast cancer cells 18, however, some studies have shown that autophagy can also inhibit cancer by protecting the integrity of the genome and inhibiting the metastasis of breast cancer cells 10. Once tumor cells form, tumor cells can use autophagy to recycle macromolecules to provide energy for the TCA cycle and substrates for biosynthesis to help them survive in adverse microenvironments. Cancer cells seem to maintain high ATP levels to meet their need for high proliferation by activating autophagy. As a result, cancer cells use autophagy to maintain a high metabolic level and thus obtain energy to help the tumor survive.

Loss of autophagy has been found in various subtypes of breast cancer, suggesting that loss of autophagy may promote tumor progression. Paradoxically, elevated levels of autophagy also promote breast cancer progression. William *et al.* found that autophagy-mediated by nuclear heterotopic action of p53 promoted the progression of breast cancer 19. Chung *et al.* found that the STK11/LKB1 signaling pathway regulates ADIPOQ/ adiponectin mediated AMPK activation, map1LC3B-II/LC3B-II release, and ULK1 (Unc-51 like kinase 1) activation, thus inducing autophagy to inhibit tumor progression 20. In contrast, gene autophagy inhibition led to significant expansion of tumor cells at both primary and metastatic sites, exhibiting invasive and premetastatic basal epithelial differentiation 10. SRC-3-AKT signaling pathway regulates autophagy of breast cancer cells to promote the proliferation of the tumor cells, and AKT further regulates mTOR signaling to form the AKT-mTOR signaling pathway that affects the poor prognosis in the patients with breast cancer 21. In addition, Jing *et al.* found that liensinine, as an alkaloid, can inhibit advanced autophagy/mitochondrial autophagy by blocking autophagosome-lysosomal fusion, thus reducing the activity of breast carcinoma cells and increasing the apoptosis of breast carcinoma cells 22. In conclusion, autophagy is closely associated with the occurrence and development of breast cancer and the generation of drug resistance. Therefore, it is very important to control its expression in the treatment of malignancy in the breast.

A major regulator of autophagy is the rapamycin (mTOR) pathway target, which is directly regulated by the signaling complex mTORC1. MTORC1 controls autophagy by controlling the phosphorylation of ATG13 and ULK1. AMPK activation occurs when intracellular energy is reduced 6, this protein kinase induces autophagy by two different mechanisms: (1) activating ATG13 and ULK1 by inhibiting the mTOR complex and (2) bypassing the mTOR signaling pathway and directly phosphorylating ULK1, VPS34, and beclin1. Cancer cells appear to activate autophagy to trigger cytotoxic and metabolic stress responses, such as hypoxia and nutrient deprivation, so they can activate autophagy to maintain cancer cells' survival. Studies have found that clinically, small molecule inhibitors can inhibit the PI3K/AKT/mTOR signaling pathway and inhibit the occurrence of autophagy, to treat breast cancer 23. Interestingly, adriamycin combined with mulandine can prevent breast cancer by inducing the expression of autophagy markers beclin-1 and LC3-II in breast carcinoma cells, reducing the expression level of p62, and inhibiting the PI3K/AKT/mTOR signaling pathway, suggesting that autophagy may be a form of cancer cell death induced by anticancer drugs 24. As reported, the p62 protein interacts with the autophagy effector protein LC3 and induces breast carcinoma cell death through the autophagy-lysosomal pathway 24. As autophagy is a survival response of tumor cells after tumor drug therapy, tumor cells enhance their sensitivity to chemotherapy drugs through autophagy 25 and realize tumor immune escape through multiple overlapping mechanisms.

In addition to autophagy-related proteins, autophagy interacts with lncRNA and signal transduction induced by lncRNA as well. Many kinds of studies have proved that lncRNA is involved in the regulation of autophagy.

## LncRNAs

Long noncoding RNAs (lncRNAs) are transcripts of more than 200 nucleotides in length 26, this kind of RNA has limited protein-coding potential and is widely found in prokaryotes and eukaryotes 27. RNA-seq studies have shown that there are thousands of unidentified lncRNAs in any given cell type, and lncRNAs may be related to developmental and tissue specificity 28. LncRNA folds into complex three-dimensional structures that interact with DNA, RNA, and proteins 29. Many molecular mechanisms of LncRNA activity have been revealed: cell cycle regulation, stem cell pluripotency 30, and regulating gene expression at different levels, including epigenetic regulation and transcriptional regulation 31. Current studies have shown that lncRNAs are closely associated with some biological processes such as development, differentiation, apoptosis, autophagy, inflammation, and cancer 32. LncRNA expression in human carcinoma cells is quite different from that in healthy cells. The first confirmed cancer-related lncRNA is HOTAIR, which is overexpressed in the tissue of breast cancer and is closely related to the occurrence and metastasis of this tumor 33. Involved in cancer and metabolic diseases, many physiological and pathological processes are highly dependent on lncRNAs. For example, some abnormal expressions of lncRNAs in cancers signal the disease progression spectrum and lncRNAs can also be used as an independent predictor of patient prognosis 18, 26, 28.

LncRNAs can be classified into cis-regulatory-lncRNAs (Cis-regulatory-lncRNAs) and trans-regulatory-lncRNAs (Trans-regulatory-lncRNAs) according to their guiding functions. Cis-regulatory-lncRNAs modify specific regions of the genome by recruiting histone complexes leading to local gene expression. It mainly includes H19, AIR, KCNQ1OT1, and XIST, among which H19 is the most widely studied in cancer and has been proved to play a role in the occurrence and inhibition of tumors 34. However, trans-regulatory lncRNAs represented by HOTAIR are upregulated in breast cancer and hepatocellular carcinoma. HOTAIR lncRNA has been found that is highly upregulated in both primary breast tumors and metastatic breast tumors and can promote cancer metastasis, and its transcription level is 2000 times higher than that of normal breast tissues 28. In cancer, when GAS5 (another trans-regulatory lncRNA) is overexpressed in breast cancer cell lines, it can induce cell apoptosis and inhibit cell proliferation. However, in human breast tumor samples, the transcription level of GAS5 are significantly reduced, which is statistically significant in stage I and II cancers 35, prompting the decrease of GAS5 expression as an early event of tumorigenesis 36.

Through the ubiquitination-autophagy pathway, lncRNA LINRIS can inhibit the proliferation of colorectal cancer cells, thus inhibiting the progression of colon cancer 37. CRNDE can increase the sensitivity of gastric cancer patients to 5-FU by inhibiting the expression of autophagy-related proteins LC-Ⅱ and LC-Ⅲ under the condition of overexpression 38. Overexpression of PVT1 in pancreatic cancer cell lines can enhance sensitivity to Gemcitabine by regulating autophagy, thus inhibiting the growth of pancreatic cancer 39. Ji *et al.* 34 demonstrated that in breast cancer cells, through the H19/SAHH/DNMT3B axis, H19 may induce autophagy activation leading to the resistance of tamoxifen in breast cancer. LncRNAs also serve as key regulators of autophagy in breast cancer. Through a review of relevant literature, the regulation of autophagy by breast cancer-related lncRNAs has been identified, 34 which may conduce to the development of new therapeutic strategies for breast cancer and more effectively target breast cancer.

## Regulation of autophagy by LncRNAs in Breast Cancer (Table 1)

Long noncoding RNAs (lncRNAs) play a significant role in the progression of tumors and are abnormally expressed in a variety of cancers 40. Current studies have found that some lncRNAs are associated with the metastasis of breast cancer and the resistance to chemotherapy drugs, and some lncRNAs are overexpressed in breast cancer to promote the progression of breast cancer 40. LncRNAs regulating autophagy through some specific mechanisms can be divided into three categories: lncRNAs bind to miRNAs as competitive endogenous RNAs (ceRNAs) to regulate miRNAs expression, thus affecting autophagy; lncRNAs may affect the expression of Cis-trans ATG genes as well. LncRNA may promote tumor progression by inhibiting autophagy-mediated apoptosis through AKT/mTOR pathway 16, 41.

### Interaction of lncRNA and autophagy in luminal A breast cancer

#### LncRNA H19

LncRNA H19 is a 2.3kb imprinted lncRNA 28. LncRNA H19 was found to be less abundant in ER-negative breast cancer than in ER-positive breast cancer. Overexpression of lncRNA H19 promotes autophagy in hormone receptor-positive breast cancer cells 42. H19 inhibits EMT (epithelial-mesenchymal transformation) of BC cells and promotes autophagy through the H19/Let-7/Lin28 signaling pathway 43. Through the H19/SAHH/DNMT3B axis, H19 may induce autophagy activation, which may help to produce tamoxifen resistance in breast cancer. Overexpression of beclin1, a key mediator of autophagy, leads to estrogen-induced desensitization of signal transduction, contributing to tamoxifen resistance in ER-positive breast cancer. Inhibition of some autophagy genes including ATG5 (autophagy-related 5), ATG7 (autophagy-related 7), and beclin1 leads to hypersensitization of tamoxifen-resistant breast cancer cells 34.

#### LncRNA HOTAIR

HOTAIR, mainly HOX antisense RNA plays an important role in chromatin dynamics and may cause transcriptional gene silence by interacting with histone modifiers 44. Bhan *et al.* found that HOTAIR overexpression promotes the development and metastasis of ER-positive breast cancer through E2 regulation 45. Since HOTAIR regulates the progression of breast cancer through the autophagy of ubiquitination and degradation of proteins in the proteasome 44, it plays a crucial role in the development of breast cancer and drug resistance in the patients with breast cancer, so it is promising to become a useful biomarker and potential therapeutic target. Studies have found that HOTAIR can regulate oral squamous cell autophagy by regulating the mTOR pathway and inhibiting cell apoptosis and cisplatin sensitivity 46. Li *et al.* found that by inhibiting the PI3K/AKT/mTOR pathway, lncRNA-HOTAIR down-regulation could effectively weaken the resistance of breast carcinoma cells to Doxorubicin 47. Xue *et al.* found in their study that HOTAIR promoted the resistance of ER-positive breast cancer to tamoxifen by up-regulating its expression in ER^+^ breast cancer patients 48, it is suggested that HOTAIR may be a useful biomarker and a potential therapeutic target.

#### LncRNA TALNEC2

Qiao *et al.* found that TALNEC2, a long non-coding RNA expressed on chromosome 2, targets p57KIP2 by binding EZH2 and is involved in the P-p38 MAPK and NF-кB pathways. It plays a carcinogenic role in luminal A and triple-negative breast cancers, and some studies have shown that TALNEC2 knockout can inhibit cell viability and community integration in luminal A and triple-negative breast cancers 49 Polycomb histone methyltransferase enhancer 2 (Polycomb histone methyltransferase enhancer of zeste homolog 2, EZH2) is considered as a key marker of invasive breast cancer 50. In addition, EZH2 regulates the NF-κB pathway by interacting with RelA and RelB complexes to regulate the development of breast cancer 51. As a major regulator in the cell cycle progression, autophagy, and apoptosis, EZH2 plays an important role in drug-resistant tumor types, suggesting that TALNEC2 may influence the progression of breast cancer and the generation of drug resistance by promoting autophagy through EZH2 52, But the specific regulation mechanism is not clear.

#### LncRNA EGOT

LncRNA EGOT may enhance the sensitivity of triple-negative breast cancer cells and Luminal A breast cancer cells to paclitaxel by upregulating ITPR1 cis and trans expression to enhance autophagosome accumulation. On the one hand, EGOT induces pre-ITPR1 accumulation by forming pre-ITPR1/EGOT dsRNA, and increases ITPR1 protein expression in a cis-manner, thus upregulating the ITPR1 level. On the other hand, two binding motifs of EGOT 2 segments (324-645 nucleotides) are enhanced the alternative splicing of trans pre-ITPR1 of EGOT in the exon 53. ITPR1 contains 59 exons and multiple GGGA/C/G motifs distributed in these exons. HnRNPH1 mediates pre-ITPR1 splicing in human cancers by binding to these GGGA/C/G motifs 54, 55. ITPR1 protein expression is decreased in an estrogen-receptor-dependent manner, and estrogen-induced GROWTH of MCF7 cells is sensitive to drug inhibitors of ITPR1. In conclusion, lncRNA enhances the sensitivity of breast cancer cells to paclitaxel therapy through enhancing autophagy.

### Interaction of lncRNA and autophagy in luminal B breast cancer

#### LncRNA ROR

As shown in many studies, LncRNA ROR can contribute to the maintenance of induced pluripotent stem cells and embryonic stem cells. In addition, lncRNA ROR (ROR, reprogramming regulator of reprogramming) is up-regulated in nasopharyngeal carcinoma, hepatocellular carcinoma, and breast cancer 56-58. Li *et al.* found that the proliferation, invasion, and migration of Luminal B breast cancer cells were inhibited by down-regulation of long non-coding RNA ROR, and the effect of tamoxifen on Luminal B breast cancer cells was reversed 59. LncRNA ROR may promote autophagy by targeting autophagy-associated proteins LC3 and beclin1, thereby promoting the progression of breast cancer and tamoxifen resistance 59. Hou *et al.* found that lncRNA ROR regulates the EMT process of breast cancer by interacting with miR-205 57. Gabriel *et al.* found that ROR could also regulate the development and metastasis of triple-negative mammary glands by interacting with miR145 60. ROR is an important target in the progression of treatment in breast cancer.

### Interaction of lncRNA and autophagy in HER-2+ breast cancer

Human epidermal growth factor receptor 2(HER-2) positive breast cancer is the most common type of breast cancer, accounting for about 15%-20% of breast cancer patients. Trastuzumab is the first-line drug in the treatment of HER-2 breast cancer 61. In 1998, Trastuzumab, approved by FDA in the United States, is designed to target and inhibit HER2 signaling in cancer cells for treating HER2-positive breast cancer 62. But about 60 percent of patients develop resistance after drug treatment, significantly reducing the clinical usefulness and efficacy of trastuzumab, which has become one of the most pressing challenges in treating breast cancer 63. So as shown in some research, lncRNA plays a vital role in some drug sensitivity of breast cancer 64.

#### LncRNA AGAAP2-AS1

ATG10 (autophagy-related 10) expression induces autophagy, leading to lncRNA AGAP2-AS1 mediated trastuzumab resistance in breast cancer, the expression of ectopic ATG10 is significantly related to lymph node metastasis and poorer prognosis in this cancer 65. Mechanically, AGAP2-AS1 is associated with ELAVL1 protein, and they are easy to form as AGAP2-AS1-ELAVL1 complex directly binding to the promoter region of ATG10, inducing enrichment of H3K27ac and H3K4me3, and ultimately activate transcription of ATG10 63. Trastuzumab-resistant breast cancer cells show higher autophagy activity, with increased LC3-Ⅱ expression and decreased P62 protein expression compared with BC parental cells, which confirms the close relationship between autophagy and tumor drug resistance. Through activating the NF-κB signaling pathway and up-regulating MyD88 expression, AGAP2-AS1 can promote the growth and trastuzumab resistance of breast tumors 66. Down-regulation of AGAP2-AS1 reverses trastuzumab resistance in HER-2 positive breast cancer 67.

#### LncRNA ZNF649-AS1

Han *et al.* found that lncRNA ZNF649-AS1 associated with PTBP1 protein induced by H3K27ac modification can promote the transcriptional activity of the ATG5 gene 64. PTB was found to be a splicing regulator that can effectively cross-link with RNA by shortwave UV light 68. AS one of the key regulators of autophagy-mediated cell death, autophagy-related gene-5 (ATG-5) is widely regarded as a protective molecular mechanism in various cancer treatments 69. Down-regulation of ZNF649-AS1 can inhibit autophagy and inhibit ATG5 expression to reverse trastuzumab resistance in breast cancer with positive HER-2.

### Interaction of lncRNA and autophagy in basal-like breast cancer

Triple-negative breast cancer, also called basal-like breast cancer, accounts for about 15%-20% of breast cancer. Due to its lack of progesterone receptor (PR), estrogen receptor (ER) and human epidermal growth factor receptor 2 (HER-2), triple-negative breast cancer patients have a worse prognosis and tumors are more aggressive, and higher mortality 70. Triple-negative breast cancer has the characteristics of strong invasion and early recurrence. Studies have proved that compared with other subtypes of breast cancer, autophagy is more intense in triple-negative breast cancer. For example, autophagy-related microtubule-associated proteins Beclin1, LC3A, and LC3B are highly expressed in this cancer cell, promoting the progression and metastasis of breast cancer 23.

#### LncRNA GAS5

Growth arrest‑specific 5 (GAS5) is known as a tumor suppressor, negatively regulating cell survival and malignancy in different cancer cell types. GAS5 lncRNA can not only inhibit the proliferation of various types of tumors, but also promote their apoptosis, and the mechanism of these cells may jointly constitute the basis of their tumor inhibition 71. LncRNA GAS5 regulates autophagy in breast cancer through the GAS5-miR-23a-ATG3 axis, and GAS5 expression is down-regulated in breast cancer. GAS5 can act as a “molecular sponge” blocking and binding miR-23a, it can positively regulate miR23A-targeting autophagy-related gene ATG3 as well, thus up-regulating GAS5 in breast cancer to inhibit tumor cell progression through autophagy 72. Mark *et al.* found in their study that lncRNA GAS5 could induce apoptosis of all breast cancer types, especially in triple-negative breast cancer 73. Li *et al.* proved in their study that GAS5 can hold up the formation, proliferation, and migration of breast cancer by mediating the autophagy-related promoter ULK1 and ULK2 (Unc-51 like kinase 2) 74.

#### LncRNA DANCR

LncRNA DANCR (Differentiation antagonist non-protein coding RNA) is carcinogenic in a variety of tumors and compared with the expression of normal issues this kind of LncRNA is higher in the tumor of breast 75. The studies have shown that miR-758-3p works as a tumor suppressor in multiple cancers, such as ovarian cancer and non-small cell lung cancer 76, 77. Research by Zhang *et al.* found that lncRNA mediating the DANCR-mir-758-3P-PAX6 molecular network can regulate apoptosis and autophagy in breast cancer. Studies have found that DANCR is negatively correlated with miR-758-3p, and down-regulation of DANCR can activate tumor suppressor miR-758-3p to play a tumor-suppressive role and promote apoptosis and autophagy of breast cancer cells: Promote the transcription and protein expression of Caspase-3, Caspase-9, Bcl-2, LC3B and ATG5 to inhibit the malignant proliferation of breast cancer cells 78.

#### LncRNA NAMPT

NAMPT-AS has potential RNA enhancer properties and can be co-transcribed with NAMPT from bidirectional promoters 79, NAMPT degradation was reduced by regulating miR-548B-3p. AS a carcinogenic lncRNA in triple-negative breast cancer, NAMPT-AS/NAMPT promotes the progression of tumors and metastasis by regulating autophagy by the mTOR pathway. Zhang *et al.* found that an increased proportion of NAMPT-AS in triple-negative breast cancers was associated with poor prognosis compared with receptor hormone-positive breast cancers. RNA-seq and Western blot analysis demonstrate that the down-regulation of the NAMPT-AS gene importantly led to the inactivation of the mTOR pathway, the up-regulation of ATG5, ATG12, and beclin, and the vital transformation of LC3-Ⅰ to LC3-II. Therefore, NAMPT-AS can inhibit the mTOR pathway and cause autophagy in TNBC cells 80. In conclusion, the NAMPT-AS/POU2F2/NAMPT and NAMPT-6 AS/miR-548B-3p/NAMPT axis activate the mTOR pathway and inhibit autophagy and apoptosis-related genes, thereby promoting the survival and invasion ability of triple-negative breast cancer cells.

#### LncRNA OTUD6B

Paclitaxel (PTX) -based combination chemotherapy remains the key to triple-negative breast cancer (TNBC) because lacking the HER-2 targeted therapy and endocrine therapy. However, chemotherapeutic resistance of up to 30% to 50% limits the effectiveness of combination drug therapy strategies, resulting in a poor prognosis 81. Direct loss, expression imbalance, or abnormal function of DDR proteins (TP53, BRCA1, ATM, etc.) all increase the risk of breast cancer, the occurrence of malignant subtypes, and the chemotherapy resistance of tumors. ATM activates autophagy through the AMPK/TSC2/mTORC1 pathway and it could also directly phosphorylate and stabilizes nuclear TP53 (tumor protein p53) to promote autophagy 82. Li *et al.* found that miR-26a-5p is a protective element to protect breast cancer from attack, in contrast, OTUD6B-AS1 and MTDH are destructive elements of breast cancer, and miR-26a-5 is down-regulated through OTUD6B-AS1/miR-26a5p/MTDH pathway. MTDH and OTUD6B-AS1 overexpression promote autophagy and DNA damage, and autophagy defects can increase the short-term sensitivity of tumor cells to chemotherapeutic drugs 83. In conclusion, OTUD6BAS1/miR-26A-5p/MTDH promotes PTX resistance in triple-negative breast cancer by upregulation of autophagy and DDR inhibition mediated genomic instability.

## The therapeutic potentials and the relevant mechanisms associated with the interference between LncRNAs and autophagy

According to current studies, lncRNA not only mediates the occurrence and development of the tumor in the breast through autophagy but also affects the metastasis and prognosis of this tumor, as well as the sensitivity of breast cancer cells to drug therapy. Autophagy activation is a response of tumor cells to chemoradiotherapy 18, 23. In most cancer patients, abnormal autophagy leads to cancerous changes that promote tumor cells' resistance to radiation and chemotherapy 25, 89. LncRNA is the main autophagy regulator affecting drug resistance to radiotherapy and chemotherapy in breast tumors 34 53. For example, Jing *et al.* found that liensinine can treat breast cancer by promoting autophagy to induce apoptosis of breast cancer cells 22. H19 contributes to the tamoxifen resistance in ER-positive breast cancer by overexpression of Beclin1, a key mediator of autophagy, leading to estrogen-induced desensitization of signal transduction 43. We found that lncRNA-mediated expression of autophagy-associated proteins promotes trastuzumab resistance in HER-2-positive breast cancer 63, 64. Li *et al.* found that the OTUD6B-AS1/miR-26a-5p/MTDH signaling pathway promotes autophagy, thereby promoting the occurrence of paclitaxel resistance in triple-negative breast cancer 83. These findings provide new insights into lncRNA-mediated autophagy as therapeutic methods for breast cancer. Abnormal tumor-associated lncRNAs in the breast system may be consistent with autophagy to identify the specific mechanisms by which tumor cells respond to drug therapy, a key area requiring further exploration.

## Limitations and future perspectives

It is the first systematic review of all evidence on the association between lncRNA and autophagy in breast tumors. A large number of specific lncRNAs-mediated-autophagy might work in the malignant transformation of breast tumors, affecting the ability of tumor cells to proliferation, apoptosis, cell cycle, migration, invasion, angiogenesis, and cell senescence. In Luminal A breast cancer, TALNEC2, GAS5, HOTAIR, H19, EGOT mediated autophagy plays a role in tumor development by influencing cancer cell development. For Luminal B breast cancer, ROR can regulate the proliferation potential of cancer cells through autophagy. For HER-2+ breast cancer, ZNF649-AS1 and AGAAP2-AS1 regulate trastuzumab resistance of breast cancer cells through autophagy, while TALNEC2 has been reported to connect with invasion and migration of breast cancer cells. However, there are some limitations in explaining the role of lncRNA in drug resistance through autophagy. First, due to limited clinical studies, the lncRNA coefficient and its clinical diagnostic, therapeutic and prognostic value still need to be further studied. Secondly, the molecular mechanisms of lncRNA-mediated autophagy, breast tumorigenesis, and drug resistance need further illustration and exploration. In addition to targeted genes, related proteins, and corresponding signaling pathways, other factors such as the microenvironment of the tumor and tumor immune checkpoints may also act a role in the pathogenesis and evolution of breast cancer. Finally, we are supposed to note that autophagy plays a dual role in cancer, not only acting as a tumor promoter but also inhibiting tumor cell development and metastasis in some breast cancer subtypes. Once the pathogenesis of breast cancer is addressed, it will be helpful to discover more useful diagnostic and prognostic biomarkers and innovative therapeutic approaches to better treat breast systemic malignancies. LncRNA-mediated autophagy has a great influence on tumor genesis, development, metastasis, and anti-tumor treatment resistance. Given this, autophagy regulation targeting the above lncRNAs may be a reliable biomarker for diagnosis and prognosis or provide a therapeutic strategy with great promise in breast cancer.

## Conclusions

In this review, lncRNA-related autophagy may be a vital molecular mechanism of breast tumor genesis and development and play a key role in breast cancer drug resistance. Up-regulation or down-regulation of lncRNAs can induce some complex procession in breast cancer through influencing autophagy. LncRNAs target genes, proteins, and different signaling pathways constituting a complex network, coordinate autophagy, affecting the carcinogenic and inhibitory effects of breast malignancies and affecting the sensitivity of cancer drug therapy.

## Figures and Tables

**Figure 1:**
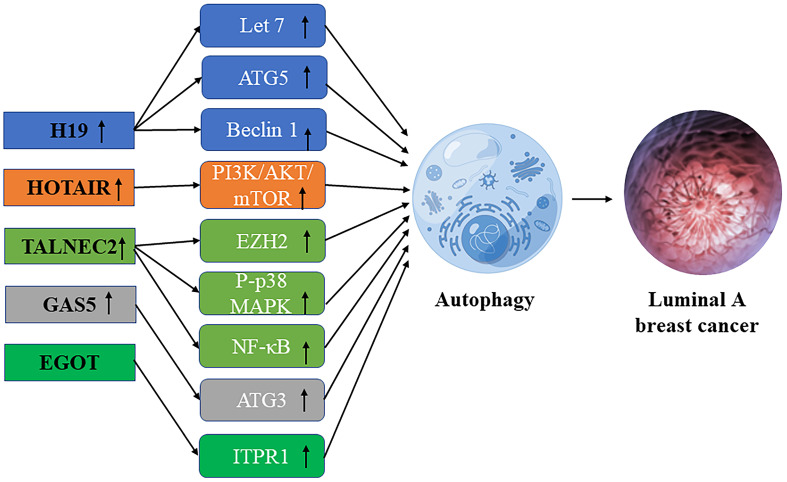
Schematic diagram of the association between lncRNA-mediated autophagy in luminal A breast cancer.

**Figure 2:**
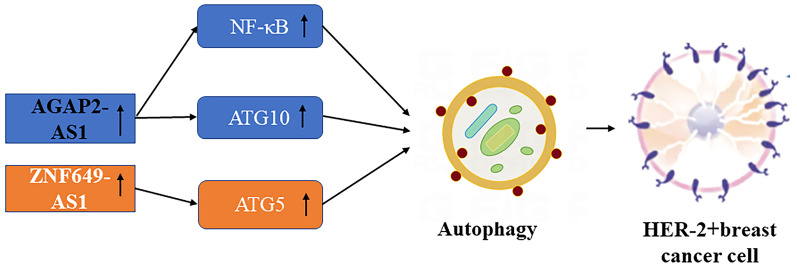
Schematic picture of the association in lncRNAs mediated autophagy of HER-2+ breast cancer.

**Figure 3:**
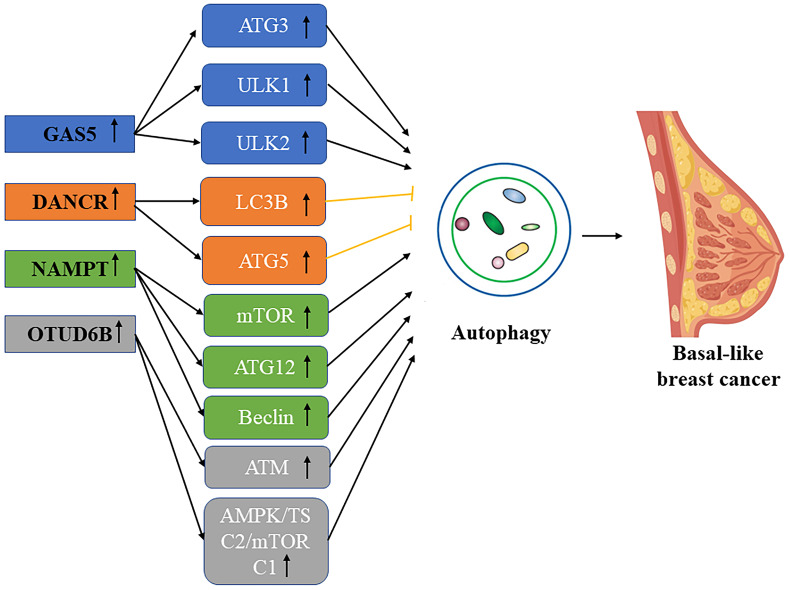
Schematic picture of the association in lncRNA mediated autophagy in basal-like breast cancer.

**Table 1:** The Role of Autophagy Mediated by LncRNAs in Initiation, Development, and Drug Resistance of Breast Cancer

Cancer Specificity	LncRNAs	Expression	Anti-/Pro autophagy	Role	References
Luminal A breast cancer	TALNEC2	Upregulated	Pro	OncoLncRNA	49
	GAS5	Downregulated	Pro	TsLncRNA	35, 36, 71-74
	HOTAIR	Upregulated	Pro	OncoLncRNA+ Promoting DR	44, 48
	H19	Upregulated	Pro	OncoLncRNA+ Promoting DR	34
	EGOT	Upregulated	Pro	Promoting DS	53
Luminal B breast cancer	ROR	Downregulated	Pro	OncoLncRNA+ Promoting DR	58
HER-2 positive breast cancer	AGAAP2-AS1	Upregulated	Pro	Promoting DR	63, 66
	EPIC1	Upregulated	--	OncoLncRNA	84
	UCA1	Upregulated	Pro	OncoLncRNA+ Promoting DR	62
	ZNF649-AS1	Upregulated	Pro	OncoLncRNA+ Promoting DR	64
Basal-like breast cancer	snaR	Upregulated	--	OncoLncRNA	85
	TALNEC2	Upregulated	Pro	OncoLncRNA	49
	OTUD6B	Upregulated	Pro	Promoting DR	83
	NAMPT	Upregulated	Anti	OncoLncRNA	80
	XIST	Downregulated	---	OncoLncRNA	86
	LUCAT1	Upregulated	---	OncoLncRNA	29
	GAS5	Downregulated	Pro	TsLncRNA	35, 36, 71-74
	HOTAIR	Upregulated	Pro	OncoLncRNA	44, 48
	WDFY3-AS2	Downregulated	---	TsLncRNA	87
	DRHC	Downregulated	---	TsLncRNA	88
	DANCR	Upregulated	Anti	OncoLncRNA	78
	EGOT	Upregulated	Pro	Promoting DS	53

DR: drug resistance; DS: drug sensitivity; BC: breast cancer; OncolncRNA: oncology lncRNA; TslncRNA: tumor suppression lncRNA.
